# Tumour-associated tenascin-C isoforms promote breast cancer cell invasion and growth by matrix metalloproteinase-dependent and independent mechanisms

**DOI:** 10.1186/bcr2251

**Published:** 2009-04-30

**Authors:** Rachael A Hancox, Michael D Allen, Deborah L Holliday, Dylan R Edwards, Caroline J Pennington, David S Guttery, Jacqueline A Shaw, Rosemary A Walker, J Howard Pringle, J Louise Jones

**Affiliations:** 1 Department of Cancer Studies and Molecular Medicine, Infirmary Close, University of Leicester, Robert Kilpatrick Clinical Sciences Building, Leicester Royal Infirmary, Leicester, LE1 5WW, UK; 2 Centre for Tumour Biology, Institute of Cancer, Barts and The London School of Medicine and Dentistry, Queen Mary University of London, Charterhouse Square, London, EC1M 6BQ, UK; 3 School of Biological Sciences, University Drive, University of East Anglia, Norwich, Norfolk, NR4 7TJ, UK

## Abstract

**Introduction:**

The stromal microenvironment has a profound influence on tumour cell behaviour. In tumours, the extracellular matrix (ECM) composition differs from normal tissue and allows novel interactions to influence tumour cell function. The ECM protein tenascin-C (TNC) is frequently up-regulated in breast cancer and we have previously identified two novel isoforms – one containing exon 16 (TNC-16) and one containing exons 14 plus 16 (TNC-14/16).

**Methods:**

The present study has analysed the functional significance of this altered TNC isoform profile in breast cancer. TNC-16 and TNC-14/16 splice variants were generated using PCR-ligation and over-expressed in breast cancer cells (MCF-7, T47D, MDA-MD-231, MDA-MB-468, GI101) and human fibroblasts. The effects of these variants on tumour cell invasion and proliferation were measured and compared with the effects of the large (TNC-L) and fully spliced small (TNC-S) isoforms.

**Results:**

TNC-16 and TNC-14/16 significantly enhanced tumour cell proliferation (*P *< 0.05) and invasion, both directly (*P *< 0.01) and as a response to transfected fibroblast expression (*P *< 0.05) with this effect being dependent on tumour cell interaction with TNC, because TNC-blocking antibodies abrogated these responses. An analysis of 19 matrix metalloproteinases (MMPs) and tissue inhibitor of matrix metalloproteinases 1 to 4 (TIMP 1 to 4) revealed that TNC up-regulated expression of MMP-13 and TIMP-3 two to four fold relative to vector, and invasion was reduced in the presence of MMP inhibitor GM6001. However, this effect was not isoform-specific but was elicited equally by all TNC isoforms.

**Conclusions:**

These results demonstrate a dual requirement for TNC and MMP in enhancing breast cancer cell invasion, and identify a significant role for the tumour-associated TNC-16 and TNC-14/16 in promoting tumour invasion, although these isoform-specific effects appear to be mediated through MMP-independent mechanisms.

## Introduction

**Figure 1:**
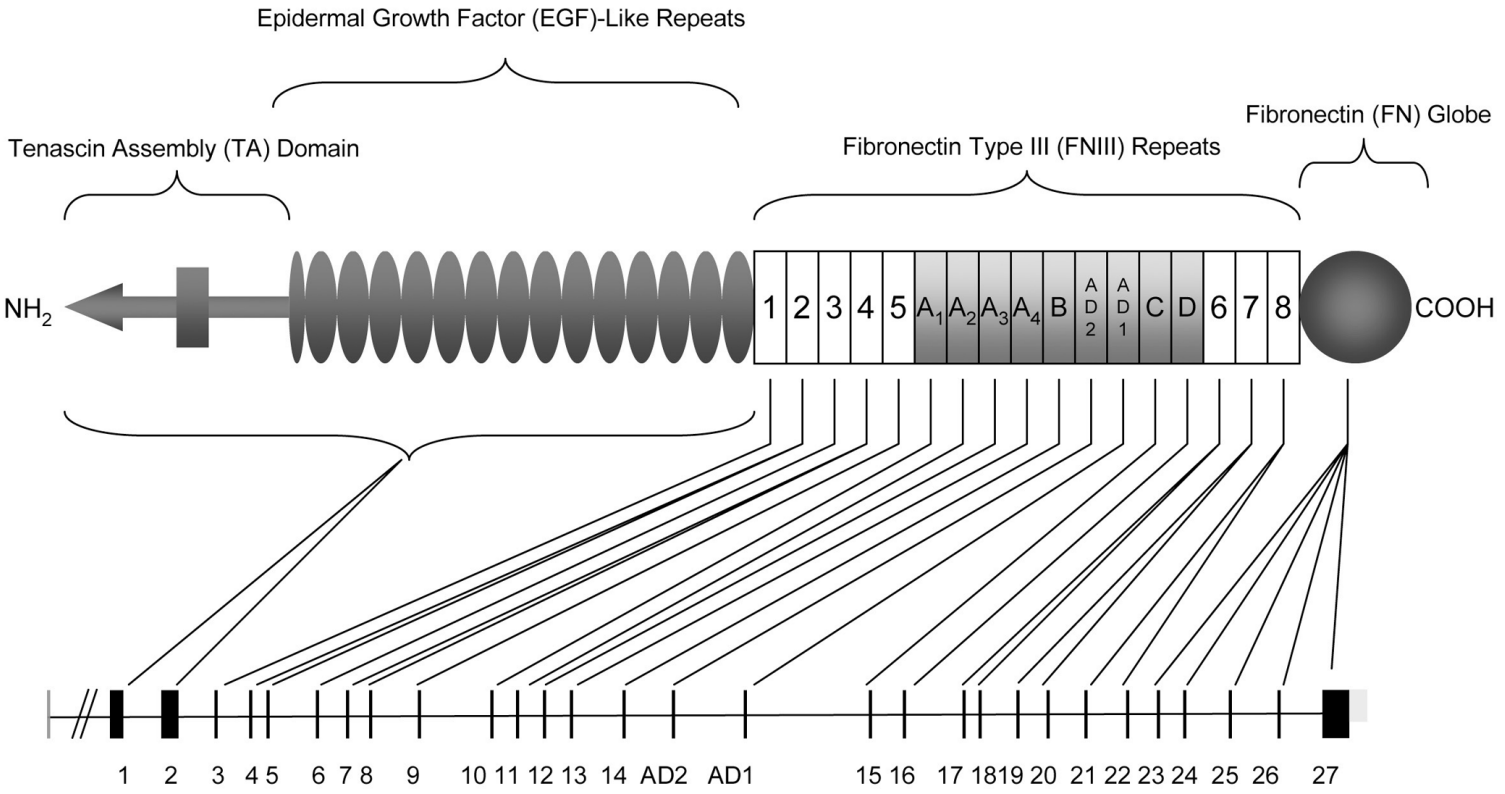
Schematic diagram of tenascin-C. The domain structure of tenascin-C comprising N-terminal tenascin assembly (TA) domain followed by 14.5 epidermal growth factor (EGF)-like repeats, the fibronectin type III (FN III)-like repeats and the carboxy fibrinogen-like domain. The FN III region consists of eight conserved repeats, designated 1 to 8, and up to nine alternatively spliced FN III repeats designated as letters A to D, AD1 and AD2 (shown in shading). The exon organisation of the tenascin-C gene is shown below.

Cellular interactions with the extracellular matrix (ECM) are critical in the modulation of cell growth, migration, invasion and tissue-specific gene expression [1-3]. The ECM around tumours differs markedly from that in normal tissues [4,5] and therefore is likely to deliver different signals to tumour cells, which will impact on their behaviour. One of the most consistent changes in the ECM of many solid tumours is up-regulation of the matrix glycoprotein, tenascin-C (TNC) [6-8]. TNC is a complex multifunctional protein, which has been shown to promote cell migration, inhibit focal contact formation, promote angiogenesis and, in some systems, act as a cell survival factor [9-11]. Each TNC subunit consists of an N-terminal tenascin assembly region, 14.5 epidermal growth factor-like (EGF) repeat domains, a variable number of fibronectin type III-like repeats (FN III) and a C-terminal fibrinogen-like domain [12] (Figure 1). Multiple isoforms of TNC can be generated through alternative splicing of nine FN III repeats between conserved repeats 5 and 6 (exons 9 and 17) at the pre-mRNA level and these may have differing effects. For example, in the developing mouse central nervous system, up to 27 distinct splice variants have been identified and are expressed in a strict temporal-spatial manner supporting a role for these variants in specific neurone-glia interactions [13]. A number of studies have shown that specific functions are mediated by distinct domains of TNC [10,11,14] and there is growing evidence to indicate that the biological function of TNC is dependent on the splicing pattern [15]. This raises the possibility that tumour-associated stroma can generate novel interactions with tumour cells through the expression of different TNC splice variants. In keeping with this, changes in the pattern of TNC isoform expression have been described in a number of malignancies [16-18], the nature of which appears to be tumour-type specific.

Previously, we have identified two TNC isoforms that consistently and specifically are up-regulated in invasive breast carcinoma as well as in a subset of pre-invasive ductal carcinoma *in-situ*: one containing exon 16 (TNC-16) the other containing exon 14 plus 16 (TNC-14/16) [19]. Up-regulation of these isoforms has also been reported in ovarian carcinoma [20]; however, their effect on tumour cell behaviour has not been established.

A further consistent change in the tumour stromal environment is up-regulation of proteolytic enzymes, particularly members of the matrix metalloproteinase (MMP) family. Overexpression of several MMPs has been described in breast cancer [21,22] and both *in-vitro *and *in-vivo *systems demonstrate a role for MMP in mediating breast cancer cell invasion [23,24]. Regulation of MMP expression and activity is complex, and may be mediated through naturally occurring tissue inhibitors of MMP (TIMP) or by direct regulation of gene expression. Several studies have indicated a role for TNC in regulating MMP gene expression [25-27].

The aim of this study was to investigate directly the effects of tumour-associated TNC-16 and TNC-14/16 isoforms on breast cancer cell behaviour and to determine whether these isoforms modulate tumour cell behaviour through regulation of MMP.

## Materials and methods

### Cell lines and primary cells

Breast cancer cell lines (MCF-7, T47D, MDA MB 231, ZR-75-1, MDA MB 468, GI101, MDA MB 436, HS578T, SkMel 28) and the human fetal foreskin fibroblast cell line hfff2 were obtained from ATCC (American Type Culture Collection, Manassas, VA, USA). Primary normal breast tissue was obtained following Ethics Approval and informed patient consent (Leicestershire, LREC 7054). Fibroblasts were isolated from reduction mammoplasty specimens and purity confirmed as previously described [28]. All cells were maintained in Dulbecco's modified Eagle's medium (DMEM) plus 2 mM L-glutamine and 10% FBS.

### TaqMan real-time PCR analysis of endogenous tenascin-C expression

Taqman real-time PCR (Applied Biosystems, Foster, CA, USA) was applied to survey different exons in breast cell lines. An inventoried assay was available for the TNC invariant exon 17/18 boundary (Applied Biosystems Taqman Assay, Hs01115654_m1) and hypoxanthine phosphoribosyltransferase 1 (HPRT1) (Applied Biosystems Taqman Assay, Hs99999909_m1) as a housekeeping gene. Primers and probe were developed in house for 9/16 and 14/16 exon boundaries. For the inventoried Taqman assays, 4 μl of cDNA (diluted 1:10) was analysed in a reaction containing 0.5 μl of probe, 0.5 μl Ultrapure water and 5 μl of 2 × Taqman Fast PCR mastermix. For the 9/16 and 14/16 assays, 3.6 μl of cDNA (diluted 1:10) was analysed in a reaction containing 0.2 μl of probe, 0.6 μl of each primer and 5 μl of 2 × Taqman Fast PCR mastermix. Normalised relative expression was determined by comparison with standard curves derived from TNC 9–16 and TNC 9–14–16 recombinant clones to correct for differences in PCR efficiency for each TNC probe set and the level of housekeeping gene expression was used to correct for any differences in cellularity.

### Generation of tenascin-C isoforms

TNC splice variant cDNAs containing either exon 16 or exons 14 and 16 were prepared by ligation and nested PCR using cloned human TNC cDNA for the large, unspliced isoform TNC-L (pNUT-HxB.L) and the truncated isoform TNC-S (pNUT-HxB.S) [29].

**Figure 2:**
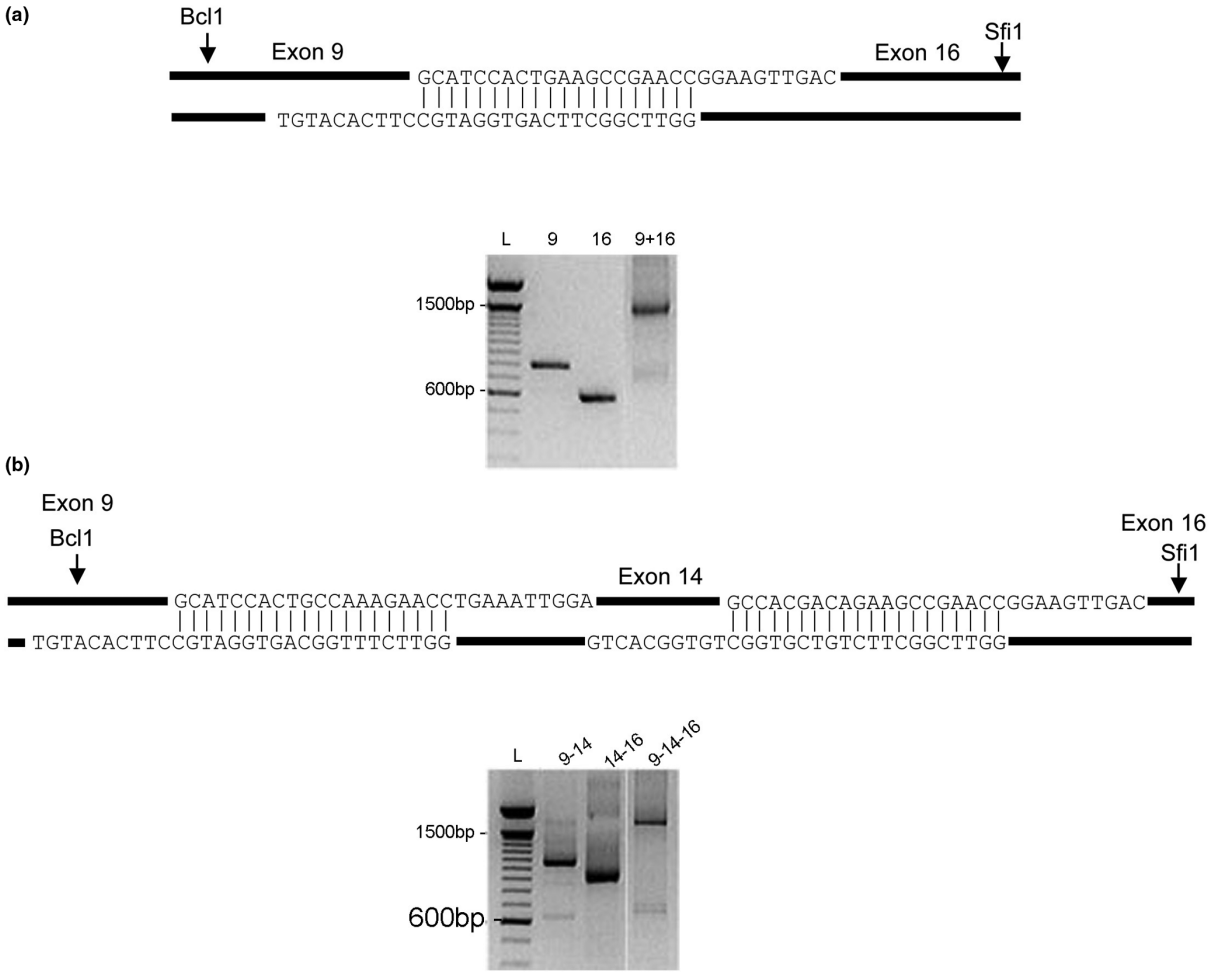
PCR-ligation strategy for generation of TNC-16 and TNC-14/16 clones. **(a) **Primers designed incorporating unique restriction sites were used to link exons 9 to 16 in a three-step polymerase chain reaction (PCR)-mediated ligation strategy. This allowed directional cloning from the Bcl1 and Sfi1 sites into the TNC-S sequence and this sequence was subsequently transferred into the mammalian expression vector pCMV script. The gel image shows exon 9 and exon 16 products and the combined 9–16 amplicon. **(b) **The same PCR-mediated ligation strategy was used to link exons 9, 14 and 16 prior to directional cloning into the TNC-S sequence for expression. The gel image shows the multi-step process used to link exons 9, 14 and 16.

Primers designed across exon boundaries 9–16, 9–14 and 14–16 were used to amplify flanking DNA to include the restriction sites *Bcl1 *and *Sfi1 *with *bcl1*ten1 and *sfi1*ten1 (Table 1 and Figure 2). These enzymes cut uniquely within exon 9 and 17 of the TNC cDNA. The amplicons were purified by Qiagen column (Sussex, UK) and ligated by nested PCR using *bcl1*ten2 and *sfi1*ten2 internal primers. The ligated DNA fragments were cloned into TOPO vector pcDNA3.1/V5/His-TOPO, screened by PCR for recombinants and sequenced. The *Bcl1 *and *Sfi1 *fragments for each spliced variant were re-cloned into TNC-S sequence in the mammalian expression vector pCMVscript and pCMV-tag4a (Invitrogen Life Science, Carlsbad, CA, USA) to produce pTNC-16 and pTNC-14/16. At each stage, all clones were confirmed by sequencing. Clones were tagged with Flag sequence to allow detection of the expressed protein.

**Table 1:** PCR primer sequences

**Primers**	**Sequence 5'-3'**
**T8-F**	CAATCCAGCGACCATCAACG
**T18-R**	CGTCCACAGTTACCATGGAG
**T9-16F**	GCATCCACTGAAGCCGAACCGGAAGTTGAC
**T9-16R**	GGTTCGGCTTCAGTGGATGCCTTCACATGT
**T9-14F**	GCATCCACTGCCAAAGAACCTGAAATTGGA
**T9-14R**	GGTTCTTTGGCAGTGGATGCCTTCACATGT
**T14-16R**	GGTTCGGCTTCTGTCGTGGCTGTGGCACTG
**T14-16F**	GCCACGACAGAAGCCGAACCGGAAGTTGAC
**Bcl1ten1**	GCCAGATCGAGGTGAAAGATGTCACA
**Bcl1ten2**	GGTGACCACCACACGCTTGGATG
**Sfi1ten1**	CTTCTGAGTCAGTGATGTTGGCTGTCACC
**Sfi1ten2**	GGCAATGGCTGGCTGCCACCT

Details of polymerase chain reaction (PCR) primers used for the generation of specific tenascin-C isoforms and to analyse expression of tenascin-C alternatively spliced forms.

### Transfection of cell populations

The breast cancer cell lines MCF-7, T47D, MDA-MB 231, MDA-MB-468 and GI101, the fibroblast cell line hfff2 and primary normal breast fibroblasts from a series of donors were transiently transfected with TNC-16, TNC-14/16, TNC-L, TNC-S or vector only using Genejuice Transfection Reagent (Novagen, Darmstadt, Germany) according to the manufacturer's instructions. In some experiments, TNC clones were transfected in combination using the same approach. Expression was confirmed by RT-PCR using the primer set 8F/18R spanning the entire alternatively spliced region (Table 1) and immunohistochemistry using the anti-Flag M2 antibody (Sigma, Dorset, UK), anti-TNC with BC-24 at 1:7500 for all isoforms and α IIIB at 1:1000 for isoforms containing exon 14 and a standard Avidin-Biotin Alkaline Phosphatase detection system. Equal transfection efficiencies for each isoform were confirmed by estimating the proportion of cells that immunostained positive.

### Western blotting

Levels of cellular and secreted TNC isoforms were determined by western blotting of transfected cell lysates and conditioned media (CM) respectively. Cells were transiently transfected and incubated for 24 hours, serum-free media was added and CM was collected after a further 48 hours. Cell lysates were harvested in gold lysis buffer containing a protease inhibitor cocktail (Sigma, Dorset, UK) 48 hours after transient transfection. Protein concentrations were quantified on a Lambda 25 UV/VIS spectrophotometer at 750 nm using the BSA protein assay, then equal amounts of protein were loaded onto 6% SDS-PAGEs and transferred to Hybond ECL nitrocellulose membrane (Amersham Biosciences, Buckinghamshire, UK). Membranes were blocked in Tris-buffered saline, 5% milk and 1% Tween for one hour and then probed for two hours with a rabbit polyclonal TNC antibody (clone H300, recognising all forms of TNC; Santa-Cruz, California, USA). A secondary antibody, donkey anti-rabbit HRP-linked IgG, 1:2000 (Amersham Biosciences, Buckinghamshire, UK), was added for one hour and blots were detected using an enhanced chemiluminescence detection kit (Amersham Biosciences, Buckinghamshire, UK).

### Analysis of tumour cell invasion

Measurement of tumour cell invasion was based on modified Boyden chamber assays as described previously [28]. To measure the direct effect of TNC isoform expression on tumour cell invasion, the transfected tumour cell population was placed in the upper chamber of the assay and a 1:1 ratio of serum-free DMEM and hfff2 CM added to the lower chamber to act as a chemotactic stimulus. CM was generated from hfff2 cells at 70% confluence, after 48 hours culture under serum-free conditions, and the media was then removed and centrifuged to remove any cell debris. To measure the indirect effect of fibroblast-associated expression of TNC on tumour cell invasion, either transiently transfected primary fibroblasts were placed in the lower well of the assay at 1 × 10^5 ^cells per well, or CM, generated from transfected fibroblasts as described above, was added to the lower chamber of the invasion assay.

The invasion assays were run over 48 hours and performed in duplicate a minimum of three times for all conditions. For each assay chamber, 20 representative fields were counted on a Leica microscope at × 200 magnification and the percentage mean invasion index (MII) calculated using the number of cells on the bottom compared with the total number of cells on the top and bottom surfaces multiplied by 100 and averaged for each experiment.

In some experiments, blocking antibody to TNC (BC-24, Sigma, Dorset, UK), IgG_1 _control (Dakocytomation, Glostrup, Denmark) or a broad-spectrum MMP inhibitor (GM6001, Chemicon, Watford, UK) were included. The TNC antibody BC-24 was desalted to remove sodium azide before use by passing through a PD-10 Sephadex G-25 column (GE Healthcare, Buckinghamshire, UK). Both BC-24 and IgG_1 _control were included in the upper and lower wells of the assay at 1 ng/ml final concentration. GM6001 was included at 10 nM.

### Analysis of tumour cell proliferation

The direct and indirect effects of TNC isoforms on tumour cell proliferation were assessed on the basis of BrdU incorporation. Tumour cells were transfected, as described above, and cultured on poly-d-lysine coated coverslips for 72 hours under serum-free conditions. BrdU was added to the media at a concentration of 5 mM for the final three hours. The cells were fixed and incorporated BrdU localised using anti-BrdU (Bu20a; Sigma, Dorset, UK) and a standard Avidin-Biotin detection system. The mean proliferation index of three assays was calculated by averaging the number of stained cells as a percentage of the total number of cells (minimum of 1000 cells over at least 10 high power fields) for each assay. To measure the indirect effect of fibroblast-associated TNC on tumour cell proliferation, fibroblast populations were transfected as previously described, the CM harvested and added to tumour cells. Proliferation was then assessed as described above.

### Analysis of matrix metalloproteinase expression and activity

#### TaqMan real-time PCR analysis of the effect of TNC isoforms on expression profile of MMP and TIMP

TaqMan real-time PCR was carried out as previously described on mRNA isolated from MCF-7 breast cancer cells and hfff2 fibroblasts transfected with different TNC clones, compared with vector-only and non-transfected control populations [30]. Analysis was performed on mRNA from three separate experiments and included measurement of 19 MMPs [3,6,7,9-18,20,22-25] and TIMP 1 to 4.

#### ELISA

For selected MMPs, levels of enzyme activity were measured by ELISA. Invasion assays were set up to include MCF-7 cells transfected with different TNC clones or CM from TNC-transfected hfff2 fibroblasts and cultured for 48 hours, as previously described. End-of-assay medium was collected from the assays, protein levels measured and equal concentrations applied to ELISA plates for MMP-1, MMP-2 and MMP-9 (Amersham, Buckinghamshire, UK). Each experiment was performed in triplicate.

#### Zymography

Levels of enzyme activity were also measured by substrate gel zymography. End-of-assay medium was collected from assays containing cells transfected with different TNC clones, as described above. SDS-PAGE substrate gels were made by incorporating Gelatin (Bloom 300, Sigma, Dorset, UK) in a 10% acrylamide separating gel at a final concentration of 1 mg/ml. To each gel, samples containing equal amounts of protein (as determined by BCA protein assay) were mixed with non-reducing sample buffer (62.5 mM Tris-HCl (pH 6.8), 10% glycerol, 2% SDS, 0.1% bromophenol blue) and added to the gel without boiling. MMP-9 recombinant pro-enzyme (Calbiochem, California, USA) and molecular weight markers were run on each gel. Following electrophoresis, gels were washed twice in 2.5% Triton X-100 for 30 minutes at 37°C to remove the SDS. Gels were incubated at 37°C overnight in developing buffer containing 50 mM Tris-HCl, 0.2 M sodium chloride, 5 mM calcium chloride and 0.02% Triton X-100. Gels were stained with 0.5% coomassie blue G250 in 30% methanol, 10% glacial acetic acid for 30 minutes and de-stained in the same solution without coomassie blue. Gelatin-degrading enzymes were identified as clear bands against the blue background of the stained gel. Images of stained gels were captured under illumination using the UVP Imagestore 5000 (Ultra-Violet Products, Cambridge, UK). Direct comparisons between separate gels were not made, because the intensity of background staining was variable. Experiments were repeated a minimum of three times.

### Statistical analysis

Statistical analysis was performed using the SPSS 12.0 statistics package (Chicago, IL, USA). For comparison between different TNC isoforms the one-way analysis of variance test was used. A Bonferroni correction was applied to all *post-hoc *analyses and a *P* value of less than 0.05 was considered significant.

## Results

### Endogenous expression of tenascin-C isoforms in breast cancer cells

**Figure 3:**
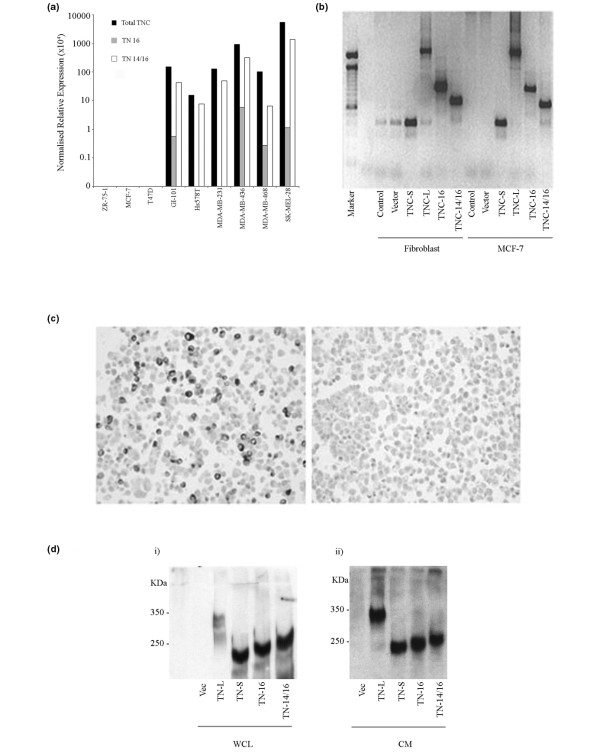
Confirmation of expression of tenascin-C isoforms. **(a) **Endogenous expression of tenascin-C (TNC) isoforms in untransfected cell lines. Normalised relative expression of TNC isoforms were determined by reverse transcriptase polymerase chain reaction (RT-PCR) using primers and probes to invariant exon 17/18 boundary (Total TNC), the 9/16 (TNC-16) and 14/16 (TNC-14/16) exon boundary for breast cell lines ZR-75-1, MCF-7, T-47D, GI-101, Hs578T, MDA-MB-231, MDA-MB-436, MDA-MB-468 and melanoma cell line SKMel-28. Relative expression was calculated by comparison with standard curves derived from TNC-9-16 and TNC 9–14–16 recombinant clones to correct for differences in PCR efficiency for each TNC probe set normalised using level of housekeeping gene expression to correct for any differences in cellularity. **(b) **RT-PCR of primary fibroblasts and MCF-7 cells transiently transfected with TNC-S, TNC-L, TNC-16, TNC-14/16 and vector-only control (vector), using primers spanning the FN III alternatively spliced region (8F/18R). This shows appropriately sized bands in each of the cell populations, with no product in vector-only and non-transfected MCF-7 controls, although there was evidence of low level expression of TNC-S in vector only and non-transfected fibroblast controls. **(c) **Immunohistochemistry for anti-Flag M2 antibody in MCF-7 cells transfected with TNC-L (left image) and for TNC (Monoclonal, BC24) in MCF-7 transfected with vector control (right image). An average transfection efficiency of 35% was determined for each TNC isoform and staining confirmed that MCF-7 cells do not express TNC. **(d) **Western blot analysis for TNC in transiently transfected MCF-7 cells. This demonstrated a single species of TNC present in the whole cell lysate (WCL; i) and conditioned media (CM; ii) of transfected cells for each isoform. TNC-S is seen as a band at about 250 kDa, with slightly larger bands detected for TNC-16 and TNC 14/16, while TNC-L is detected at about 350 kDa.

Real-time PCR for endogenous TNC isoform expression was performed on a series of breast cancer cell lines. This showed that MCF-7, T47D and ZR-75-1 cell lines do not express detectable levels of TNC or its isoforms, while GI-101, Hs578T, MDA MB 231, MDA MB 436 and MDA MB 468 cells all express TNC. Furthermore, all express TNC-14/16 and GI-101, MDA 436 and MDA MB 468 also express TNC-16 (Figure 3a).

### Expression of tenascin-C isoforms in breast cancer cells and fibroblasts

The breast cancer cells MCF-7, T47D, MDA MB 231, MDA MB 468 and GI101, the hfff2 fibroblast cell line and a series (n = 5) of primary normal breast fibroblasts were transiently transfected with clones for TNC-S, TNC-L, TNC-16 and TNC-14/16 and vector only controls. Expression of specific isoforms was confirmed by RT-PCR (Figure 3b), and protein expression was confirmed by immunohistochemistry to the Flag tag (Figure 3c), which demonstrated an average transfection efficiency of 35% for each isoform. No native TNC was detected in MCF-7 cells (Figure 3c, right image). The level of expression in transfected MCF-7 cells was equivalent to that seen endogenously in MDA-MB-231 cells (unpublished data).

Western blotting of cell lysates (Figure 3d, i) and CM (Figure 3d, ii) demonstrated equivalent levels of TNC protein expression from each of the clones, and confirmed the presence of TNC.

### Direct effects of tenascin-C isoforms on tumour cell behaviour: effect on invasion and proliferation

**Figure 4:**
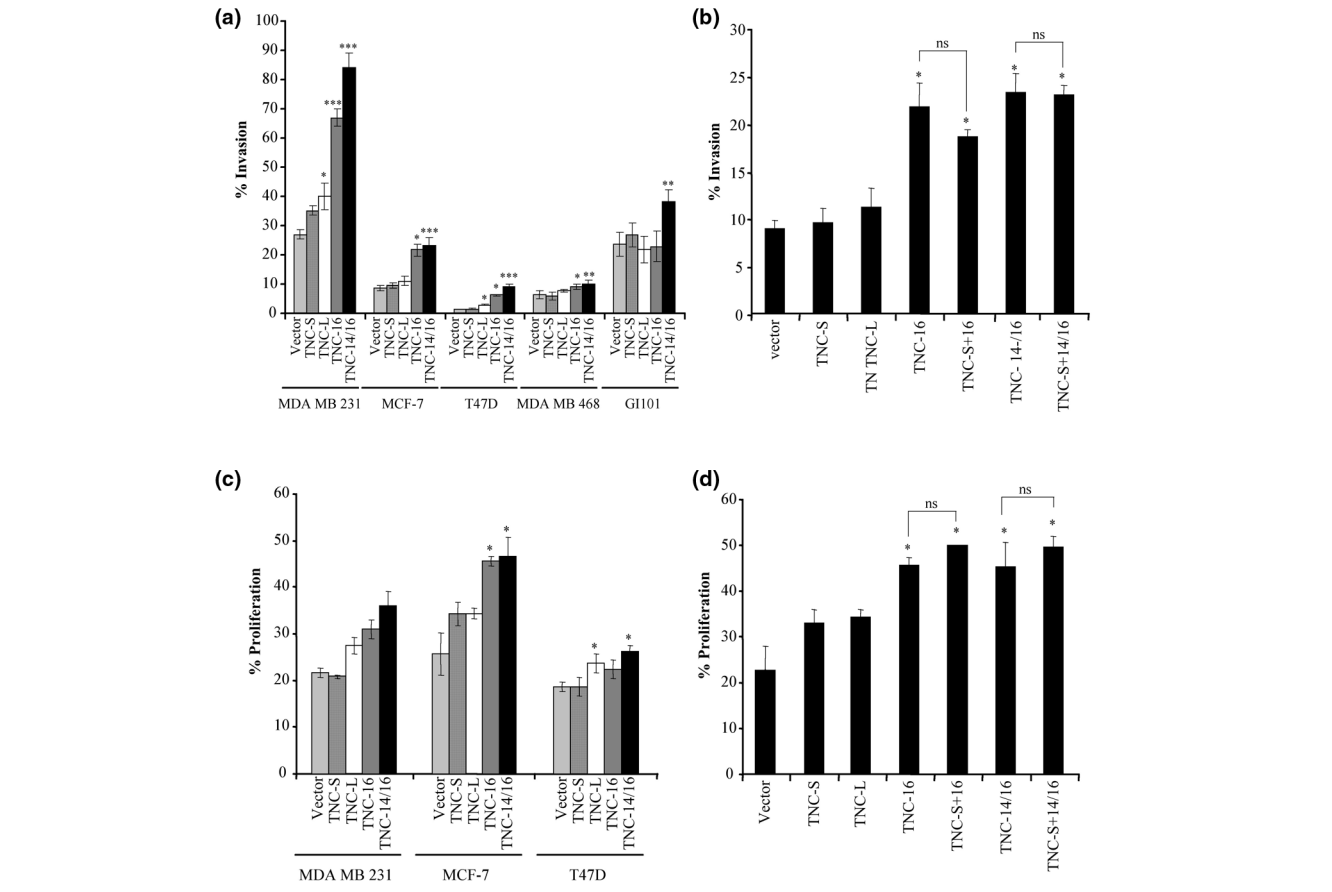
Direct effects of tenascin-C on tumour cell invasion. **(a) **Invasion of MDA MB 231, MCF-7, T47D, MDA MB 468 and GI101 cell lines transfected with four different tenascin-C (TNC) isoform constructs and the vector alone. MDA-MB-231 and T47D cells transfected with TNC-L show a higher mean invasion index (MII) than controls (*P *< 0.05). All cell lines exhibited significantly higher MII with TNC-16 and TNC-14/16 compared with the vector alone (*P < 0.05, **P < 0.01, ***P < 0.001), other than GI101, which showed significantly higher invasion with TNC-14/16 only (*P *= 0.01). **(b) **Effect of co-transfection of TNC-S with either TNC-16 or TNC-14/16 isoforms on MCF-7 cell invasion. Both TNC-16 and TNC-14/16 led to an increased MCF-7 MII (*P < 0.01) compared with vector alone and other isoforms. However, there was no further enhancement in MII when the MCF-7s were co-transfected with TNC-S and either TNC-16 or TNC-14/16 (ns = not significant). **(c) **Proliferation of MDA MB 231, MCF-7 and T47D cell lines transfected with four different TNC isoforms constructs and the vector alone. MBA-MB-231 cells exhibit no significant changes in proliferation when transfected with any of the isoforms. MCF-7 and T47D both show increased proliferation when transfected with TNC-14/16 compared with vector alone (*P < 0.05). MCF-7 exhibited an increase in proliferation with TNC-16 compared with vector alone, whereas T47Ds did not show any changes with TNC-16 but did have a significant (*P *< 0.05) increase in proliferation with TNC-L. **(d) **Effect of co-transfection of TNC-S with either TNC-16 or TNC-14/16 isoforms on MCF-7 cell proliferation. Both TNC-16 and TNC-14/16 lead to an increased MCF-7 proliferation (*P < 0.01) compared with vector alone and other isoforms. However, there was no further enhancement in proliferation when the MCF-7s were co-transfected with TNC-S and either TNC-16 or TNC-14/16 (ns = not significant). In all cases, the bars indicate the mean of at least three experiments with standard errors shown.

The breast cancer cell lines exhibited different levels of invasion. The MCF-7, T47D and MDA MB 468 cell lines showed low level invasion (Mean Invasion Index (MII) of 8%, 2% and 6%, respectively) whereas the MDA-MB-231 and GI101 cell lines exhibited high invasion (MII of 27% and 23% respectively; Figure 4a). For cell lines transfected with either the vector alone or TNC-S there was no significant change in the MII compared with the untreated cells (Figure 4a). TNC-L transfectants in MDA-MD-231 and T47D showed increased MII over baseline levels (*P *= 0.005 and *P *= 0.011, respectively). However, the highest levels of invasion were observed for each of the cell lines when transfected with TNC-14/16 (*P *< 0.001 for MDA-MB-231, *P *< 0.001 for MCF-7, *P *= 0.001 for T47D, *P *= 0.005 for MDA-MB-468, *P *= 0.01 for GI101) and (except for GI101) TNC-16 (*P *< 0.001 for MDA-MB-231, *P *= 0.004 for MCF-7 and *P *= 0.002 for T47D). The TNC-14/16 isoforms also showed significantly higher MII than the TNC-L transfectants, *P *< 0.01 for all cell lines. The MII was also significantly higher with TNC-16 vs. TNC-L for MDA MB 231 (*P *= 0.008), MCF-7 (*P *= 0.004), T47D (*P *= 0.009) and GI101 (*P *= 0.01) cells but not MDA MB 468 (Figure 4a).

In human breast carcinoma tissues, TNC-16 and/or TNC-14/16 are not always detected alone, but frequently are seen in combination with TNC-S [19]; therefore MCF-7 cells were co-transfected with TNC-S and either TNC-16 or TNC-14/16. The results showed that although TNC-16 and TNC-14/16 both enhanced MCF-7 cell MII, there was no additional effect of co-expression of TNC-S (Figure 4b).

Increased breast cancer cell proliferation was observed in all cell lines over-expressing TNC-L, TNC-16 and TNC-14/16. This reached significance in MCF-7 cells for both TNC-16 and TNC-14/16 (*P *= 0.023 and *P *= 0.002, respectively), and in T47D cells for TNC-L and TNC-14/16 (*P *= 0.025 and *P *= 0.011, respectively; Figure 4c). A similar effect was seen in MCF-7 cells co-transfected with TNC-S and either TNC-16 or TNC-14/16 with no additive effect over the single isoforms (Figure 4d).

### Effect of fibroblast-associated tenascin-C expression on tumour cell invasion

**Figure 5:**
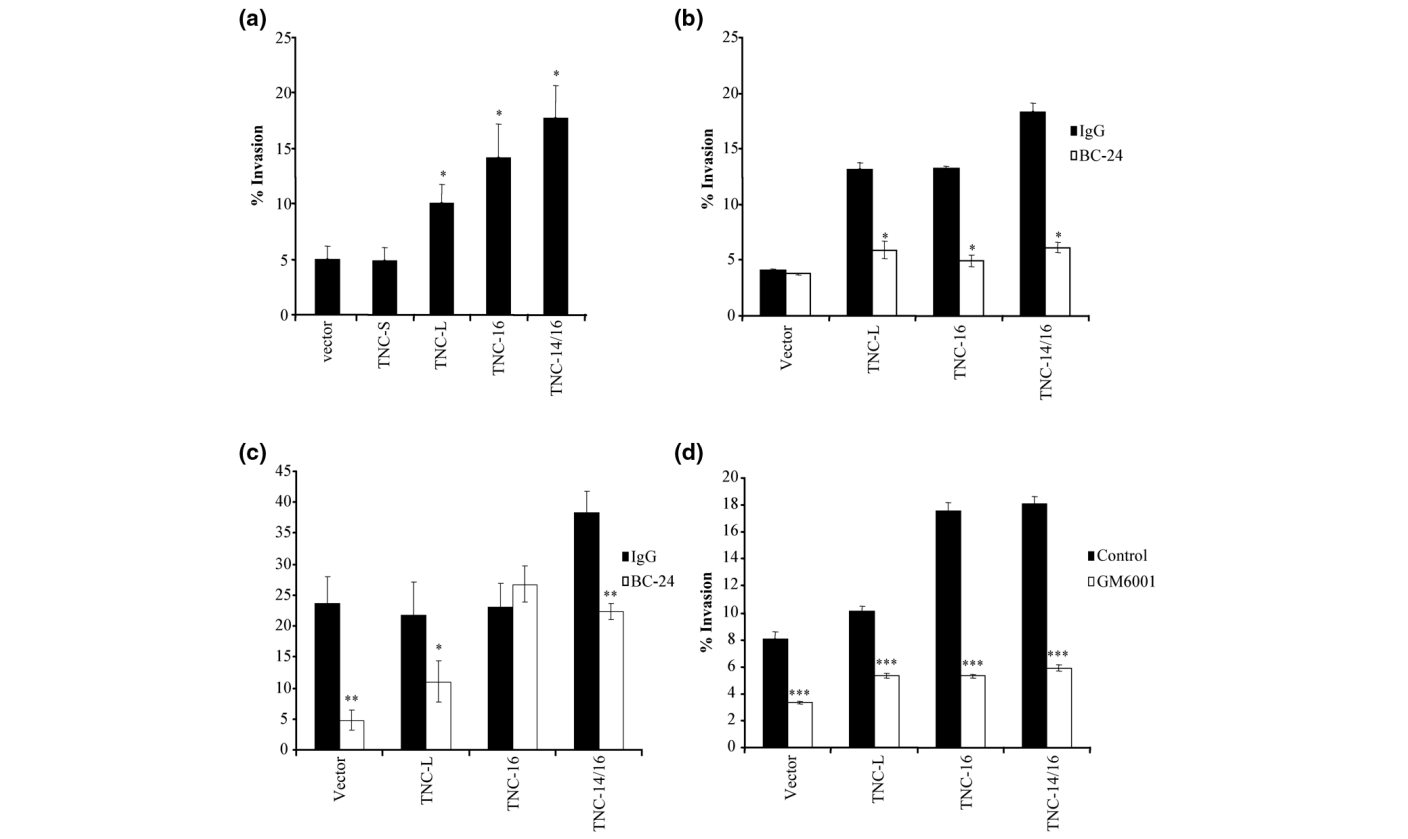
Effect of fibroblast-associated tenascin-C expression on MCF-7 tumour cell invasion. **(a) **Mean invasion index (MII) for MCF-7 tumour cell co-cultured with primary breast fibroblasts (n = 5) transfected with tenascin-C (TNC) isoforms constructs or vector alone. Fibroblasts expressing TNC-16 or TNC-14/16 significantly increased MII compared with TNC-L (*P *= 0.05 and *P *= 0.001, respectively), TNC-S and vector alone (***P < 0.001). Higher MII was also seen with fibroblast expressing TNC-L (*P *< 0.001) compared with TNC-S and vector alone. Bars indicate the mean of five donors, each measured in triplicate. **(b) **MCF-7 cell MII using conditioned media from primary breast fibroblasts transfected with TNC isoforms constructs or the vector alone in the presence of blocking TNC mouse monoclonal antibody BC-24 or equivalent IgG control. The blocking antibody significantly reduced MII (*P < 0.05) independent of the TNC isoform. In all cases, the bars indicate the mean of three experiments with standard errors shown. **(c) **GI101 cell MII using conditioned media from fibroblasts transfected with TNC isoforms constructs or the vector alone in the presence of blocking TNC mouse monoclonal antibody BC-24 or equivalent IgG control. The blocking antibody significantly reduced MII (*P < 0.05, **P < 0.01) in all the cases except for TNC-16. In all cases, the bars indicate the mean of three experiments with standard errors shown. **(d) **MCF-7 cells transfected with different TNC isoforms or vector only all exhibited significant reduction in MII in the presence of the matrix metalloproteinase inhibitor GM6001 (***P < 0.001). Bars indicate mean of three experiments.

The major source of TNC in breast carcinomas is the peri-tumoural stroma [19], therefore we analysed the effect of fibroblast-associated TNC isoform expression on tumour cell invasion. Primary breast fibroblasts isolated from normal breast tissue were transfected with individual TNC isoforms and co-cultured in the lower chamber of invasion assays with MCF-7 breast cancer cells. Standard invasion assays were then carried out for each TNC isoform with vector-only controls. There was some variability between donors in the capacity of fibroblasts to promote tumour cell invasion (data not shown). However, for all donors, a significantly higher MCF-7 MII was observed in the presence of fibroblasts over-expressing TNC-L, TNC-16 or TNC-14/16 (*P *< 0.001, Figure 5a) but not in the presence of TNC-S. Furthermore, a higher MII for MCF-7 cells was seen with fibroblasts over-expressing TNC-16 or TNC-14/16 compared to TNC-L (*P *= 0.05 and *P *= 0.001, respectively). These results reflect the pattern seen when the tumour cells themselves over-express TNC, and a similar pattern also was seen in the presence of CM from transfected primary fibroblasts (data not shown).

TNC is secreted by cells [31], so we investigated whether the effect of fibroblast-associated TNC isoforms was mediated directly through soluble TNC. CM from transfected fibroblasts was added to the lower well of invasion assays containing MCF-7 cells (Figure 5b) or GI101 cells (Figure 5c) with addition of blocking antibody to TNC (1 ng/ml BC-24) or equivalent IgG_1 _control. This demonstrated inhibition of tumour cell invasion to control levels, (cells transfected with vector only) or lower for all MCF-7 transfected lines in the presence of TNC antibody BC-24 and for all GI-101 cells other than those transfected with TNC-16. This suggests that the pro-invasive effect of TNC-transfected fibroblast CM is mediated directly through secreted TNC.

### Effect of tenascin-C isoforms on MMP expression and activity

To address whether TNC might mediate enhanced invasion through up-regulation of MMP, invasion assays were performed with the addition of the broad-spectrum MMP inhibitor GM6001. This demonstrated a reduction in invasion of MCF-7 cells under all conditions, both control and those transfected with TNC isoforms (Figure 5d), indicating that invasion is dependent on MMP activity but is not specifically TNC related.

MMP expression was investigated by TaqMan real-time RT-PCR, ELISA for total enzyme activity and zymography for relative levels of active and latent enzyme in MCF-7 cells and hfff2 fibroblasts transiently transfected with TNC isoform constructs and control. A thorough analysis of 19 MMP family members and all four TIMP members in MCF-7 [See Additional data files 1, 2 and 3] and hfff2 (data not shown) revealed that MMP-13 and TIMP-3 were upregulated two to fourfold relative to the vector but this was independent of the TNC isoform. None of the other MMPs or TIMPs analysed by real-time PCR exhibited significant changes in expression in response to TNC or were expressed at such low levels that any variation in expression was likely to be non-significant [See Additional data files 1, 2 and 3] MMPs 1 and 2 were undetectable in MCF-7 cells and did not show any variation in hfff2 cells (data not shown). ELISAs demonstrated no significant change in the activity levels of MMP-1 or 2, while MMP9 was not detected in MCF-7 or hfff2 by ELISA [See Additional data file 4]. Zymography showed no change in MMP9 activation levels between different clones when transfected into hfff2 [See Additional data file 5] or MCF-7 cells (data not shown).

## Discussion

The stromal microenvironment plays a critical role in determining tumour cell behaviour [32-34]. Changes in the matrix protein composition and extensive remodelling by proteolysis are two key mechanisms by which the microenvironment can promote tumour progression [6,11,13,23].

Tenascin-C is a complex ECM protein that frequently is up-regulated in the matrix around solid tumours [6,7]. The diverse effects of TNC are mediated, in part, through the existence of multiple alternatively spliced isoforms, which appear to be regulated in a strict temporal-spatial manner, implying a complex structure-function relationship [13,35]. We previously have shown that, in addition to a quantitative change in TNC in breast cancer, there is a consistent change in the pattern of TNC isoform expression, with induction of two additional isoforms – TNC-16 and TNC-14/16 – rarely detected in normal resting breast [28]. In this study analysis of a series of breast cancer cell lines demonstrated expression of TNC-16 and/or TNC-14/16 in all oestrogen receptor (ER) negative lines characterised by more aggressive behaviour [36], but not in the ER-positive cell lines. We demonstrated that each of these TNC isoforms increased breast cancer cell invasion significantly and also enhanced tumour cell proliferation. The largest, unspliced isoform TNC-L has been associated with a motile phenotype most frequently [10,18,22] and, in keeping with this, was found to promote invasion in this study. However, the effect was significantly less than obtained with expression of the tumour-associated TNC-14/16 isoforms and, in all but GI101 cells, also with TNC-16. The fully spliced, adult-type TNC-S had no effect on tumour cell invasion or proliferation, consistent with its role as a component of normal basement membrane in many tissues [22].

Inclusion of domains within the alternatively spliced FN III region may alter the biological function of TNC through a number of mechanisms. Some of the domains introduce new adhesion motifs, for example, domain D (exon 14) contains a binding site for α7β1 integrin [37], domains B and D interact with the cell adhesion molecule F3/contactin [38], while the A1-4/BD region binds to the cell surface receptor annexin II [39]. Domains A3 and D also contain sites susceptible to proteolytic cleavage [40] leading to generation of TNC fragments that have been implicated in tumour progression [41]. Finally, the alternatively spliced FN III region can also modify the interaction of TNC with other ECM proteins, specifically fibronectin [42]. Thus, the precise structure of the TNC molecule determines the final biological effect.

The mechanism by which TNC-16 and TNC-14/16 promote breast cancer cell invasion and proliferation is unclear, although it does appear to require direct interaction between the tumour cell and the protein, because the pro-invasive effect was blocked by TNC antibody. Furthermore, the effect of stromal-derived isoforms appear to be mediated through a direct effect on the tumour cell rather than modification of fibroblast function because the promotion of invasion by TNC-transfected fibroblast CM was completely abrogated by anti-TNC antibodies, suggesting that in transwell assays the soluble TNC within the CM is acting as a chemoattractant mediating in the effect on invasion. These data imply a novel adhesive interaction between TNC-16, TNC-14/16 and the breast cancer cells, although the nature of this currently is unclear. It is unlikely that this interaction replicates the described interaction between domain D (exon 14) and α7β1 on neurites [37], because these breast cancer cells do not express α7 integrin (unpublished data). It is interesting that TNC-L, which contains both exons 14 and 16, does not promote invasion to the same extent as the smaller isoforms. This is reminiscent of the interplay between domains identified in fibronectin. The central Arg-Gly-Asp (RGD)-containing 120FN fragment of fibronectin induces MMP expression in rabbit synovial fibroblasts, but regions outside this domain inhibit this induction [43]. Thus it is plausible that additional domains in the TNC-L protein counteract the invasion-promoting effect of exons 14 and 16. Further work using peptide fragments to investigate the interactions between different domains of TNC would help dissect these functions.

The actions of MMPs have been implicated in many aspects of cancer progression including invasion, as a result of proteolytic cleavage of MMPs and cell adhesion molecules [24,44], and may influence tumour growth via release of matrix-bound growth factors [45]. The importance of MMP activity in the systems used in this study is demonstrated by the significant reduction of tumour cell invasion by a broad-spectrum MMP inhibitor. This led us to investigate whether TNC-16 and TNC-14/16 isoforms up-regulate MMP expression or activity. A relationship between TNC and MMP expression has been implied in a number of studies, generally as a result of correlation of expression levels or co-localisation in tissue studies [46,47]. There also is emerging evidence that TNC can modulate MMP levels directly. Tremble and colleagues [27] showed that TNC could increase MMP-1, 3 and 9 expression in rabbit synovial fibroblasts, but only in collaboration with fibronectin and not if added as a soluble protein. Kalembeyi and colleagues [25] demonstrated up-regulation of MMP-9 expression in mouse mammary carcinoma cells in response to exogenous TNC, while a recent study reported that the invasion-promoting effect of TNC on glioma cells is mediated through up-regulation of MMP-12 [26].

In this study, an extensive analysis of MMP and TIMP expression revealed that TNC up-regulates MMP-13 and TIMP-3, but that this is not specific to TNC-16 or TNC-14/16 isoforms, with TNC-S, which does not generate enhanced tumour invasion over control levels, also elevating expression levels to a similar extent. Both MMP-13 and TIMP-3 have been implicated in breast cancer [48]. Thus their induction by TNC may be of relevance *in vivo*, although in the systems used in this study, they do not appear to be specifically mediating the tumour-promoter effect of TNC-16 and TNC-14/16. Furthermore, no change in MMP activity, as revealed by zymographic analysis, was seen in relation to different TNC isoforms. Thus, although MMP activity is clearly required for invasion, these results suggest that TNC-16 and TNC-14/16 mediate their action through MMP-independent mechanisms. TNC has been shown to up-regulate MMP in previous studies [25-27]; however, this difference may be due to a number of reasons: species- and cell-type differences; the collaborating effect of other MMPs; a three-dimensional environment compared with the monolayer system used in this study [26]; and, of key importance, the nature of the TNC protein being investigated.

The precise nature of the TNC protein employed in previous studies is unclear. However, if obtained commercially, as it appears to be in at least one of the studies, [26] it is likely to represent a mixture of isoforms predominantly of the largest splice variants and these could have quite distinct functions from the isoforms used in the present study. Although we did not demonstrate any interaction between TNC-S and TNC-16 or TNC-14/16 in co-transfection experiments, we did not investigate the additional influence of TNC-L as this was less frequently detected in the breast carcinoma tissues we analysed [19].

It is possible, and quite likely, that different TNC isoforms exert their biological effects through different mechanisms. Thus while it appears that many TNC species can influence MMP expression, it is possible that TNC-16 and TNC-14/16 mediate their effects on invasion and proliferation by signalling through tumour cell adhesion receptors rather than through MMP up-regulation. It is clear, however, that in the system described in this study MMPs are a key requirement for tumour invasion. The interplay of different factors in mediating a process as complex as invasion is not surprising, and the inter-dependence of multiple factors in this process has previously been reported [31].

## Conclusions

This study demonstrates a dual requirement for TNC and MMP activity in breast cancer cell invasion and it confirms that the tumour-associated TNC-16 and TNC-14/16 isoforms significantly enhance this invasive process, even above the TNC-L isoform traditionally associate with cell migration. The almost universal and high level of expression of these isoforms in breast carcinomas coupled with their largely tumour-restricted distribution make them a plausible therapeutic target, a strategy that already is being employed for tumour-restricted TNC isoforms in other systems [49,50].

## Abbreviations

BSA: bovine serum albumin; CM: conditioning medium; ECM: extracellular matrix; EGF: epidermal growth factor; ELISA: enzyme-linked immunosorbent assay; ER: oestrogen receptor; DMEM: Dulbecco's modified Eagle's medium; FBS: fetal bovine serum; FN III: fibronectin type III-like repeats; HPRT1: hypoxanthine phosphoribosyltransferase 1; MII: mean invasion index; MMP: matrix metalloproteinase; PCR: polymerase chain reaction; RT-PCR: reverse transcriptase polymerase chain reaction; TIMP: tissue inhibitor of matrix metalloproteinases; TNC: tenascin C; TNC-16: tenascin C with only exon 16 of variable region; TNC 14/16: tenascin C with only exons 14 and 16 of variable region; TNC-L: tenascin C largest splice variant; TNC-S: tenascin C smallest splice variant.

## Competing interests

The authors declare that they have no competing interests.

## Authors' contributions

JHP and JS created the TNC isoform expression vectors. RH carried out invasion assays, proliferation assays and immunostaining and blotting. MA carried out invasion assays and zymography. DH and MA carried out ELISA. DG carried out QPCR for TNC isoforms. DE, CP and MA performed experiments for MMP QPCR. JHP, JLJ, JS and RA conceived of the study, participated in its design and coordination and helped to draft the manuscript. RH, DH and MA performed statistical analysis. All authors read and approved the final manuscript.

## Supplementary Material

Additional file 1 A Powerpoint file containing a figure showing real-time polymerase chain reactions for matrix metalloproteinase (MMP) 3, 8, 9, 10, 11, 13, 14 and 15 expression. The mean level of MMP gene expression relative to 18s in control MCF-7 vs isoform transfected MCF-7 cells. MMP1 and 2 did not provide any signal. Click here for file

Additional file 2 A Powerpoint file containing a figure showing real-time polymerase chain reactions for matrix metalloproteinase (MMP) 16, 17, 18/19, 2, 23, 24, 25 expression. The mean level of MMP gene expression relative to 18s in control MCF-7 vs isoform transfected MCF-7 cells. Click here for file

Additional file 3 A Powerpoint file containing a figure showing real-time polymerase chain reactions for matrix metalloproteinase (MMP) 27, 28 and tissue inhibitor of matrix metalloproteinase (TIMP) 1 to 4 expression. The mean level of MMP and TIMP gene expression relative to 18s in control MCF-7 vs isoform transfected MCF-7 cells. Click here for file

Additional file 4 A Powerpoint file containing a figure showing ELISA to determine secreted levels of matrix metalloproteinase (MMP) 1, 2 and 9. ELISA analysis was carried out on conditioned media from transfected MCF-7 and hfff2 cells for MMP 1, 2 and 9; however, no signal was determined for MMP9 in either cell line. Click here for file

Additional file 5 A JPG file containing a figure showing a zymogram for matrix metalloproteinase (MMP) expression. Hfff2 fibroblasts were transiently transfected with the four tenascin isoforms, after 24 hours the media was changed to serum free and conditioned for 48 hours. Equal protein concentrations were applied to a 10% SDS-PAGE containing gelatin. The first lane contains recombinant MMP9 as a molecular weight marker and control. Click here for file
