# Supplementary material for: Tumour-associated tenascin-C isoforms promote breast cancer cell invasion and growth by matrix metalloproteinase-dependent and independent mechanisms
Source: Breast Cancer Res. 2009 Apr 30;11(2):R24. doi: 10.1186/bcr2251 (PMC2688953; doi:10.1186/bcr2251)
Supplement: Additional file 1 — A Powerpoint file containing a figure showing real-time polymerase chain reactions for matrix metalloproteinase (MMP) 3, 8, 9, 10, 11, 13, 14 and 15 expression. The mean level of MMP gene expression relative to 18s in control MCF-7 vs isoform transfected MCF-7 cells. MMP1 and 2 did not provide any signal. [file bcr2251-S1.ppt]

## Slide 1
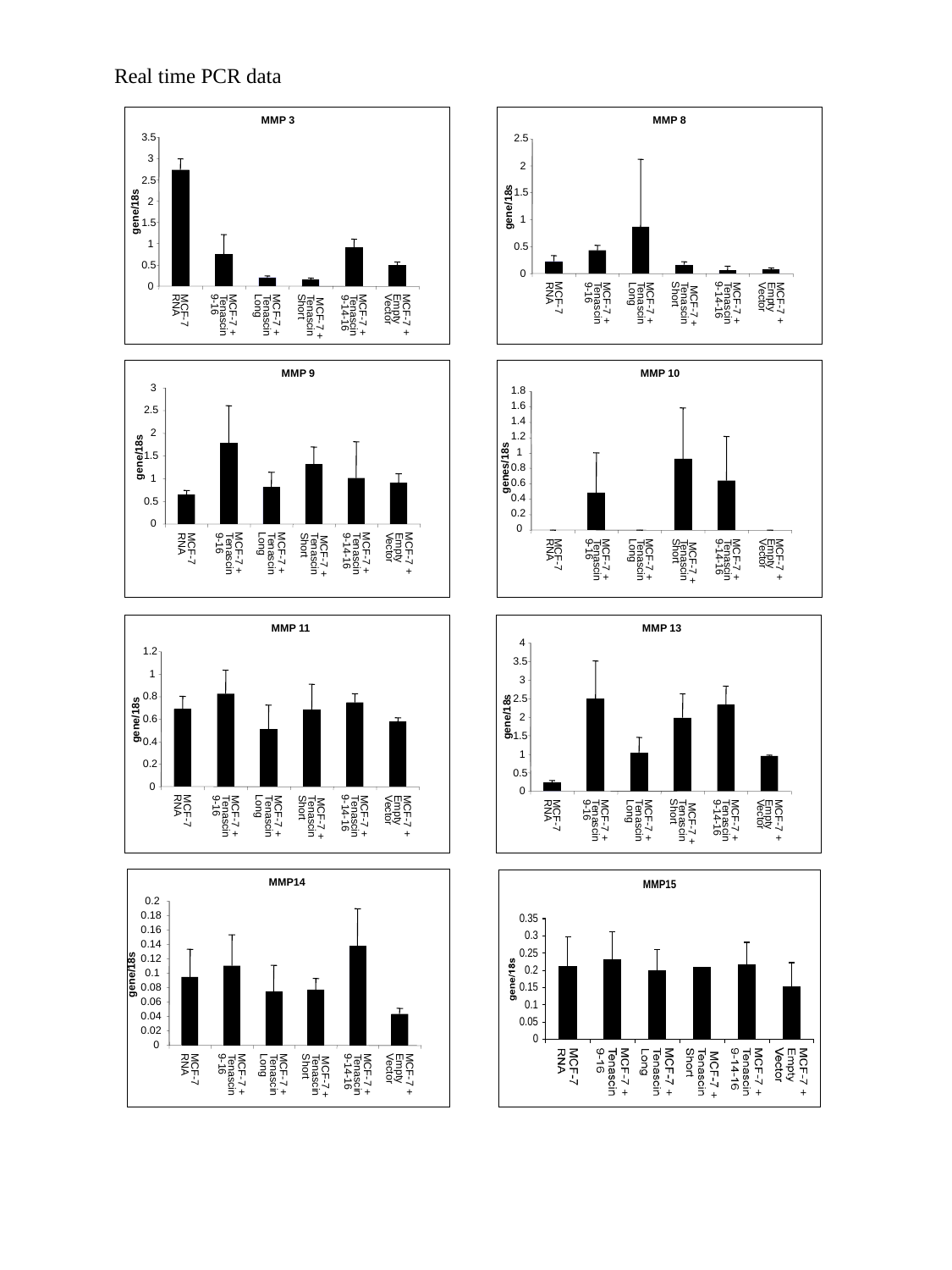

Real time PCR data
MMP 3
3.5
3
2.5
2
gene/18s
1.5
1
0.5
0
9-16
RNA
Long
Short
Empty
Vector
MCF-7
9-14-16
MCF-7 +
MCF-7 +
MCF-7 +
MCF-7 +
Tenascin
Tenascin
Tenascin
Tenascin
 MCF-7 +
MMP 8
2.5
2
1.5
gene/18s
1
0.5
0
9-16
RNA
Long
Short
Empty
Vector
MCF-7
9-14-16
MCF-7 +
MCF-7 +
MCF-7 +
MCF-7 +
Tenascin
Tenascin
Tenascin
Tenascin
 MCF-7 +
MMP 9
3
2.5
2
1.5
gene/18s
1
0.5
0
9-16
RNA
Long
Short
Vector
Empty
MCF-7
9-14-16
MCF-7 +
MCF-7 +
MCF-7 +
MCF-7 +
Tenascin
Tenascin
Tenascin
Tenascin
 MCF-7 +
MMP 10
1.8
1.6
1.4
1.2
1
0.8
genes/18s
0.6
0.4
0.2
0
9-16
RNA
Long
Short
Empty
Vector
MCF-7
9-14-16
MCF-7 +
MCF-7 +
MCF-7 +
MCF-7 +
Tenascin
Tenascin
Tenascin
Tenascin
 MCF-7 +
MMP 11
1.2
1
0.8
0.6
gene/18s
0.4
0.2
0
9-16
RNA
Long
Short
Empty
Vector
MCF-7
9-14-16
MCF-7 +
MCF-7 +
MCF-7 +
MCF-7 +
Tenascin
Tenascin
Tenascin
Tenascin
 MCF-7 +
MMP 13
4
3.5
3
2.5
2
gene/18s
1.5
1
0.5
0
9-16
RNA
Long
Short
Vector
Empty
MCF-7
9-14-16
MCF-7 +
MCF-7 +
MCF-7 +
MCF-7 +
Tenascin
Tenascin
Tenascin
Tenascin
 MCF-7 +
 MMP14
0.2
0.18
0.16
0.14
0.12
0.1
gene/18s
0.08
0.06
0.04
0.02
0
9-16
RNA
Long
Short
Empty
Vector
MCF-7
9-14-16
MCF-7 +
MCF-7 +
MCF-7 +
MCF-7 +
Tenascin
Tenascin
Tenascin
Tenascin
 MCF-7 +
