# Supplementary material for: Tumour-associated tenascin-C isoforms promote breast cancer cell invasion and growth by matrix metalloproteinase-dependent and independent mechanisms
Source: Breast Cancer Res. 2009 Apr 30;11(2):R24. doi: 10.1186/bcr2251 (PMC2688953; doi:10.1186/bcr2251)
Supplement: Additional file 2 — A Powerpoint file containing a figure showing real-time polymerase chain reactions for matrix metalloproteinase (MMP) 16, 17, 18/19, 2, 23, 24, 25 expression. The mean level of MMP gene expression relative to 18s in control MCF-7 vs isoform transfected MCF-7 cells. [file bcr2251-S2.ppt]

## Slide 1
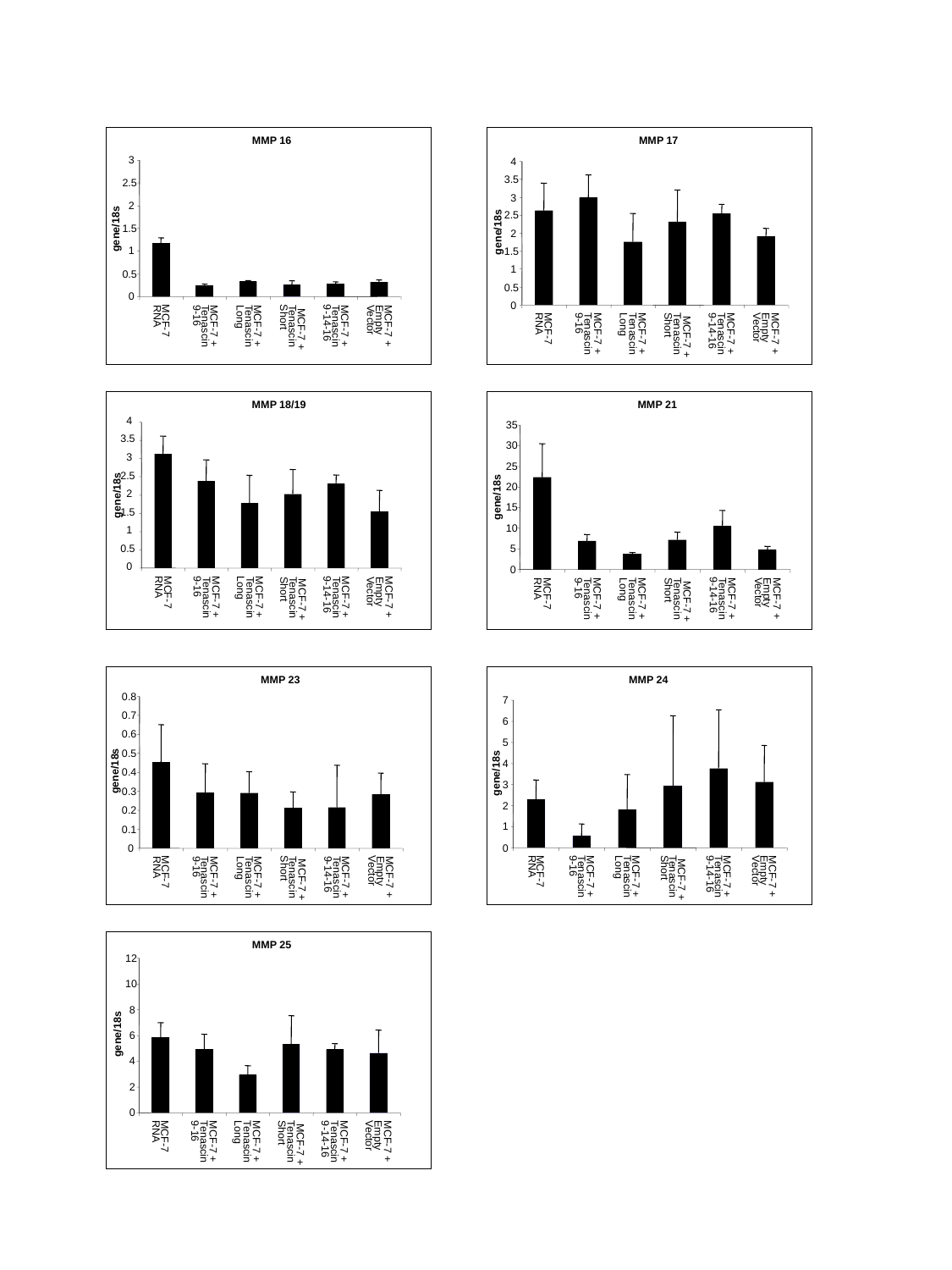

MMP 16
3
2.5
2
1.5
gene/18s
1
0.5
0
9-16
RNA
Long
Short
Vector
Empty
MCF-7
9-14-16
MCF-7 +
MCF-7 +
MCF-7 +
MCF-7 +
Tenascin
Tenascin
Tenascin
Tenascin
 MCF-7 +
MMP 17
4
3.5
3
2.5
gene/18s
2
1.5
1
0.5
0
9-16
RNA
Long
Short
Empty
Vector
MCF-7
9-14-16
MCF-7 +
MCF-7 +
MCF-7 +
MCF-7 +
Tenascin
Tenascin
Tenascin
Tenascin
 MCF-7 +
MMP 18/19
4
3.5
3
2.5
2
gene/18s
1.5
1
0.5
0
9-16
RNA
Long
Short
Empty
Vector
MCF-7
9-14-16
MCF-7 +
MCF-7 +
MCF-7 +
MCF-7 +
Tenascin
Tenascin
Tenascin
Tenascin
 MCF-7 +
MMP 21
35
30
25
20
gene/18s
15
10
5
0
9-16
RNA
Long
Short
Vector
Empty
MCF-7
9-14-16
MCF-7 +
MCF-7 +
MCF-7 +
MCF-7 +
Tenascin
Tenascin
Tenascin
Tenascin
 MCF-7 +
MMP 23
0.8
0.7
0.6
0.5
gene/18s
0.4
0.3
0.2
0.1
0
9-16
RNA
Long
Short
Empty
Vector
MCF-7
9-14-16
MCF-7 +
MCF-7 +
MCF-7 +
MCF-7 +
Tenascin
Tenascin
Tenascin
Tenascin
 MCF-7 +
MMP 24
7
6
5
4
gene/18s
3
2
1
0
9-16
RNA
Long
Short
Empty
Vector
MCF-7
9-14-16
MCF-7 +
MCF-7 +
MCF-7 +
MCF-7 +
Tenascin
Tenascin
Tenascin
Tenascin
 MCF-7 +
MMP 25
12
10
8
gene/18s
6
4
2
0
9-16
RNA
Long
Short
Empty
Vector
MCF-7
9-14-16
MCF-7 +
MCF-7 +
MCF-7 +
MCF-7 +
Tenascin
Tenascin
Tenascin
Tenascin
 MCF-7 +
