# Supplementary material for: Tumour-associated tenascin-C isoforms promote breast cancer cell invasion and growth by matrix metalloproteinase-dependent and independent mechanisms
Source: Breast Cancer Res. 2009 Apr 30;11(2):R24. doi: 10.1186/bcr2251 (PMC2688953; doi:10.1186/bcr2251)
Supplement: Additional file 4 — A Powerpoint file containing a figure showing ELISA to determine secreted levels of matrix metalloproteinase (MMP) 1, 2 and 9. ELISA analysis was carried out on conditioned media from transfected MCF-7 and hfff2 cells for MMP 1, 2 and 9; however, no signal was determined for MMP9 in either cell line. [file bcr2251-S4.ppt]

## Slide 1
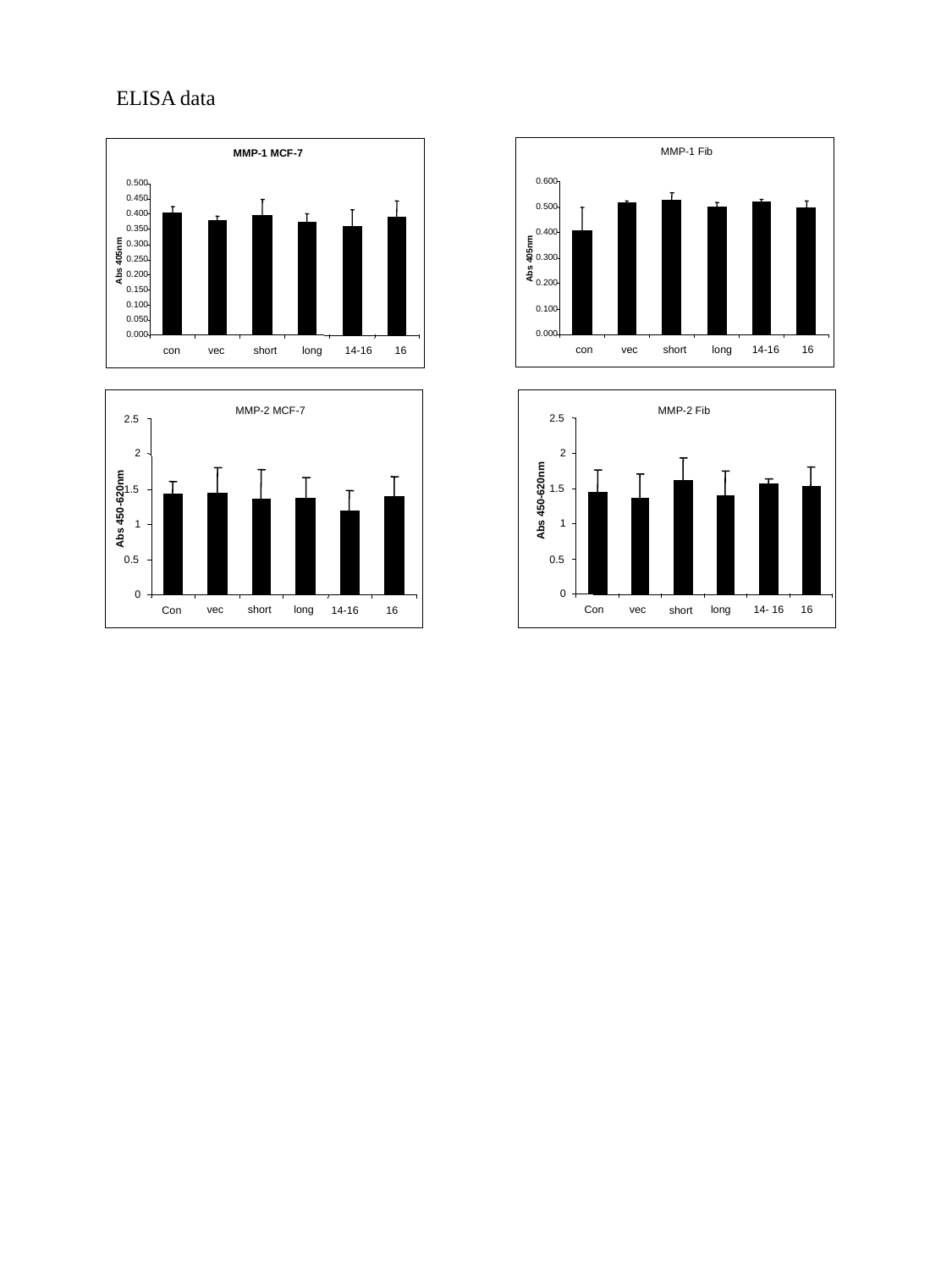

ELISA data
MMP-1 Fib
MMP-1 MCF-7
0.600
0.500
0.450
0.500
0.400
0.350
0.400
0.300
0.300
Abs 405nm
0.250
Abs 405nm
0.200
0.200
0.150
0.100
0.100
0.050
0.000
0.000
 con
 vec
 short
 long
 14-16
 16
con
vec
 short
 long
 14-16
 16
2.5
2
1.5
Abs 450-620nm
1
0.5
0
vec
short
long
14-16
16
Con
2.5
2
1.5
Abs 450-620nm
1
0.5
0
 Con
 vec
 long
14- 16
 16
short
MMP-2 MCF-7
MMP-2 Fib
