# Supplementary figures and images for: Tumour-associated tenascin-C isoforms promote breast cancer cell invasion and growth by matrix metalloproteinase-dependent and independent mechanisms
Source: Breast Cancer Res. 2009 Apr 30;11(2):R24. doi: 10.1186/bcr2251 (PMC2688953; doi:10.1186/bcr2251)

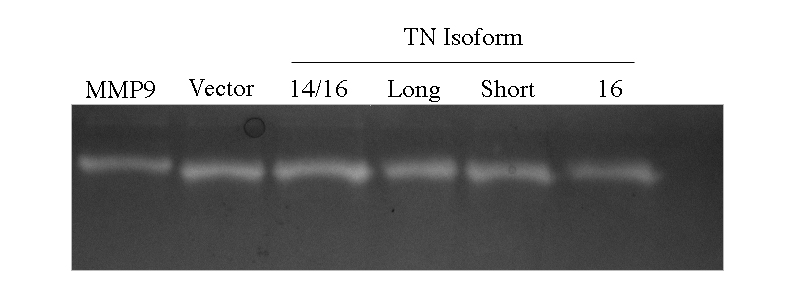

Supplement: Additional file 5 — A JPG file containing a figure showing a zymogram for matrix metalloproteinase (MMP) expression. Hfff2 fibroblasts were transiently transfected with the four tenascin isoforms, after 24 hours the media was changed to serum free and conditioned for 48 hours. Equal protein concentrations were applied to a 10% SDS-PAGE containing gelatin. The first lane contains recombinant MMP9 as a molecular weight marker and control. [file bcr2251-S5.jpeg]
